# Large-Scale Public Transcriptomic Data Mining Reveals a Tight Connection between the Transport of Nitrogen and Other Transport Processes in *Arabidopsis*

**DOI:** 10.3389/fpls.2016.01207

**Published:** 2016-08-11

**Authors:** Fei He, Abhijit A. Karve, Sergei Maslov, Benjamin A. Babst

**Affiliations:** ^1^Biological, Environmental and Climate Sciences Department, Brookhaven National LaboratoryUpton, NY, USA; ^2^Purdue Research FoundationWest Lafayette, IN, USA; ^3^Department of Bioengineering, Carl R. Woese Institute for Genomic Biology, National Center for Supercomputing Applications, University of Illinois at Urbana-ChampaignUrbana, IL, USA; ^4^Arkansas Forest Resources Center, The University of Arkansas at MonticelloMonticello, AR, USA

**Keywords:** coexpression network, NRT, nitrate transporter, big data, *Arabidopsis*, public expression data

## Abstract

Movement of nitrogen to the plant tissues where it is needed for growth is an important contribution to nitrogen use efficiency. However, we have very limited knowledge about the mechanisms of nitrogen transport. Loading of nitrogen into the xylem and/or phloem by transporter proteins is likely important, but there are several families of genes that encode transporters of nitrogenous molecules (collectively referred to as N transporters here), each comprised of many gene members. In this study, we leveraged publicly available microarray data of *Arabidopsis* to investigate the gene networks of N transporters to elucidate their possible biological roles. First, we showed that tissue-specificity of nitrogen (N) transporters was well reflected among the public microarray data. Then, we built coexpression networks of N transporters, which showed relationships between N transporters and particular aspects of plant metabolism, such as phenylpropanoid biosynthesis and carbohydrate metabolism. Furthermore, genes associated with several biological pathways were found to be tightly coexpressed with N transporters in different tissues. Our coexpression networks provide information at the systems-level that will serve as a resource for future investigation of nitrogen transport systems in plants, including candidate gene clusters that may work together in related biological roles.

## Introduction

Nitrogen (N) is often the most limiting nutrient for plant growth. The US produces more than 10 million tons of nitrogen fertilizer annually in order to increase the output of agriculture (Russell et al., 2009). The process of making those fertilizers is energy intensive, and excessive fertilization leads to environmental pollution due to leaching and run-off of N into rivers and oceans. Understanding the mechanisms of nitrogen utilization in plants will provide guidance to improve nitrogen use efficiency of crop plants, which will reduce fertilizer and energy costs of agriculture, and help protect our environment (Canfield et al., 2010).

N is usually taken up by roots from the soil as nitrate or ammonium, or sometimes organic forms, such as amino acids (Masclaux-Daubresse et al., 2010). Nitrate and ammonium may be assimilated into organic forms in the roots or leaves through the glutamine synthetase-GOGAT (GS-GOGAT) cycle, and may be utilized or stored where synthesized, or translocated to other tissues (Masclaux-Daubresse et al., 2010). For example, in many crop plants, when there is limited N availability, N is translocated from older leaves to younger leaves higher on the stem that typically receive more direct sunlight and are less likely to be shaded than older leaves (Diaz et al., 2008). Transport between different plant tissues may occur by loading N into the xylem or phloem, where it moves with the bulk flow of the xylem or phloem sap, respectively. Some of the genes that control N transport have been identified, but other components remain to be identified and our understanding of the system is incomplete.

Nitrogen in different chemical forms can be transported by different gene families, such as amino acid transporters (AAT), nitrate transporters/peptide transporters (NPF, formerly called NRT1 and PTR), and NRT2 (Tsay et al., 2007; Léran et al., 2014), ammonium transporters (AMT), amino acid-polyamine-choline transporters (APC), and amino acid/auxin permeases (AAAP) (Williams and Miller, 2001), which we will collectively call “N transporters” here for brevity. Uptake of nitrate from soil is perhaps the best understood aspect of N transport in plants. Plants have evolved two types of transporters, high and low affinity, for the uptake of nitrate from soil at low and high concentrations, respectively, and those transporters are induced or repressed accordingly (Orsel et al., 2002). After uptake, nitrate may be assimilated to organic forms in the roots, or loaded into the xylem by NPF7.3 (formerly NRT1.5) for transport from roots to leaves, where it may be assimilated or stored in the vacuole (Wang Y. -Y. et al., 2012). Although plants often recycle this valuable nutrient from the old leaves to new organs (Wang Y. -Y. et al., 2012), the genes involved in recycling have not yet been fully determined (Tegeder, 2012). Also, the system responsible for translocation of N from leaves to reproductive organs has not been fully elucidated, although some components have been identified. For example, the amino acid permeases, such as AAP2 and AAP6, mediate transfer of amino acids from the xylem to the phloem, impacting N and protein content of seeds, and other silique and seed-localized transporters, such as AAP1 and NPF2.12 (formerly NRT1.6) mediate seed development and filling (Almagro et al., 2008; Tegeder, 2012). Environmental conditions in the soil may vary drastically, including the level of nitrate availability, and factors such as nitrogenous metabolite concentrations in specific tissues, circadian rhythm, sucrose, and pH may play a role in regulating N utilization (Gojon et al., 2009; Krouk et al., 2010). Thus, we expect coordinated coregulation of genes that act together as a system in response to these varying conditions.

Microarray technology has provided the power to measure mRNA abundance efficiently and affordably, and has been used to study N utilization in plants (Wang et al., 2003; Bi et al., 2007; Krouk et al., 2009). Generally, the mRNA samples from plants with no or limited N and sufficient N supply are compared in order to find the differentially expressed genes (DEG), which are considered to be the candidates involved in N utilization. A series of computational studies have been performed based on the microarray measurements in order to investigate the gene networks underlying these processes (Gutiérrez et al., 2007a,b; Stokes et al., 2008; Nero et al., 2009). For instance, Nero et al. integrated 76 microarray samples from five labs and identified a gene network module which may be responsive to nitrate. Unlike traditional research focusing on one or a few genes, these studies provided a genome-wide view of nitrogen utilization, which may help us better understand the mechanisms at a higher level (Ruffel et al., 2010). The idea behind those studies generally is that genes with a similar expression pattern across many samples may be functionally related (Rhee and Mutwil, 2014). Plant transcriptomic data have accumulated in the past decade and more than 30,000 expression profiling samples for *Arabidopsis* are stored in NCBI GEO (Barrett et al., 2013). Despite the abundance of the data, making sense of those public data remains challenging (Rung and Brazma, 2013). In order to detect the stable coexpression relationships, microarray datasets from different labs have been combined to calculate the correlation coefficient between two expression profiles (Kim et al., 2001; Stuart et al., 2003; Atias et al., 2009; Mao et al., 2009; Wang S. et al., 2012). Often correlations between different genes may depend on the specific cellular context (De la Fuente, 2010), for example cancer vs. non-cancer cells (Anglani et al., 2014). The problem with combining microarray data from many different experiments is that context-specific relationships may be missed.

We applied context-specific coexpression analysis, first for a subset of genes involved in nitrogen transport, 17 genes (15 NRTs and of 2 other families that encode channels that transport nitrate), and then on a larger scale for 170 genes potentially involved in the nitrogen transport system in *Arabidopsis* from multiple gene families (Table S1). Unlike previous computational works, we processed each GEO dataset independently in order to capture context-specific regulation relating to nitrogen transport. We analyzed microarray datasets from 320 studies done by different labs, including not only microarray data generated for the study of nitrogen but also microarray data from studies unrelated to nitrogen. Candidate genes and pathways that might be involved or associated with nitrogen transport were discovered, which will guide further experimental studies.

## Results

### Differential expression across experiments indicates context-specific gene functionality within a tissue

**Figure 1:**
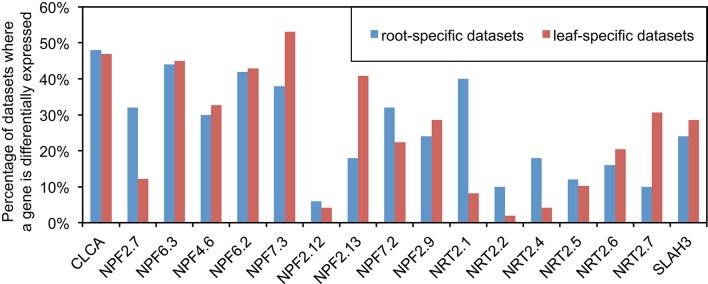
**Comparison of differential expression between roots and leaves for N transporters**. ANOVA followed by FDR was utilized to detect differential expression between replicated groups in each GEO datasets (*p* < 0.01). There are 50 datasets which contain root samples only and 49 datasets which contain leaf samples only among 371 datasets collected for this study (see Table S2 for detail).

Although both ammonium and nitrate can be used by plants, nitrate is the major form of nitrogen in many soils (Chrispeels et al., 1999). We focused initially on 15 NRTs and two other genes that encode channels that transport nitrate (Figure 1), each of which has some experimental evidence of its function (Wang Y. -Y. et al., 2012). We first explored differential expression, since those genes are believed to be regulated to respond to certain signals and hence they may play a role during the studied biological process (Tarca et al., 2006). Among the 371 published *Arabidopsis* expression series datasets we collected from GEO, 50 datasets are root-specific and 49 datasets are leaf-specific (see Table S2). For each leaf- and root-specific dataset, we identified the DEG and tallied the number of experiments in which each gene was differentially expressed (see Section Materials and Methods). Comparing the differential expression events between roots and leaves was suggestive of the function of some genes. For example, NPF2.7 (formerly NAXT1) was differentially expressed in more than 30% of root-specific datasets but in only about 12% of leaf-specific datasets (Figure 1), suggesting a context-specific function of NPF2.7 in roots. Previous studies have demonstrated that NPF2.7 is involved in the excretion of nitrate from roots of *Arabidopsis* (Segonzac et al., 2007). Also, our results show that NPF2.13 (formerly NRT1.7) was differentially expressed in only about 20% of roots but in about 40% of leaves (Figure 1). It has been reported that NPF2.13 is involved in translocation of nitrate from old leaves to young leaves (Fan et al., 2009). Furthermore, NRT2.1 and NRT2.2 are differentially expressed in roots twice as much as in leaves (Figure 1), which corresponds to their roles in the uptake of nitrate from soil (Wang Y. -Y. et al., 2012). We must caution that there are caveats to using this approach as an indicator of functionality. For example, NRT2.4 has much higher differential expression in roots than in leaves, which is consistent with its function in root nitrate uptake (Kiba et al., 2012). However, NRT2.4 has a second role, relating to the loading of nitrate into the phloem in shoots, which would have been missed by the differential expression approach alone. These examples suggest that comparing the relative level of responsiveness of genes in particular tissues is one approach that could be combined with other approaches to focus functional genomics studies of gene networks, especially for large gene families like NRT (e.g., cytochrome P450s, glycosyltransferases, glycoside hydrolases, etc.). Additionally, the measure of plasticity in expression provided by this analysis is suggestive of the degree to which a gene's function within a tissue is dependent on context and conditions.

### Coexpression analysis across 320 datasets identified related metabolic processes and possible pathway members relating to transport of nitrogen

**Figure 2:**
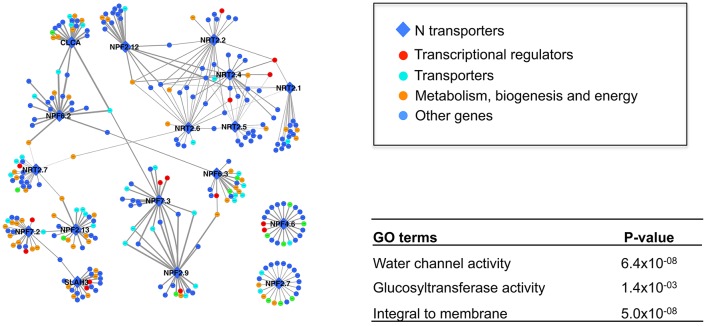
**The coexpression network of 17 N transporters**. Only the top 20 coexpressed genes for each N transporter were included. The width of the edge is corresponding to the weight of average coexpression among 320 GEO datasets. A high resolution version of this figure and the coexpression weight can be found in Supplemental Materials (Figure S3 and Table S3).

When building a coexpression network, Pearson Correlation Coefficient (PCC) is often used to measure the weight of correlation between two expression profiles. The challenge in selecting a cutoff to define what elements to include is that the minimum value of PCC that is significantly different from zero (i.e., no correlation) heavily depends on the sample size. For example, at the significance level 0.05, the minimum PCC is 0.6 when the sample size is 10, and 0.2 when the sample size is 100. Generally speaking, there is no standardization of the cutoff amongst studies, and it may vary dramatically (Jordan et al., 2004; Van Noort et al., 2004; Wang S. et al., 2012). Although the *p*-value of correlation can be used as cutoff between datasets of different sample size (Ponomarenko et al., 2013), it is tricky to calculate an average value using *p*-value. Since coexpression networks are intended to make complex systems understandable to the human intellect, others have included only a small number of most highly correlated genes (e.g., the top 20 genes or top 0.1%) (Kim et al., 2001; Bergmann et al., 2004). We utilized a similar strategy to focus attention on the most highly correlated network members (See Section Materials and Methods). A network was constructed using the top 20 coexpressed partners for our 17 focal genes (i.e., 15 NRT and 2 channels) (Figure 2). The weight (i.e., PCC) for each GEO dataset was calculated independently and the average value of all datasets was used to measure the strength of coexpression between a pair of genes. Unlike using a combined meta-dataset where the transient/context-specific signals may be swamped, those relationships are more likely to be captured by our method (Usadel et al., 2009).

Many of the 17 N transporters were coexpressed with several other N transporters, showing the potential functional association among those genes. In order to test whether this coexpression network makes biological sense, GO enrichment analysis was performed to detect over-represented functional categories after removing all of the 17 genes from the network (Figure 2). Interestingly, “water channel activity” was over-represented (*p* = 6.4 × 10^−8^), which indicates that transport of water and transport of nitrate may be under coordinated transcriptional control. Other than those 17 genes, there are various transporters, metabolic enzymes, and transcriptional regulators in this network (Figure 2), some of which appear likely to have a relationship with the nitrogen transport system, based on their known functions. For example, NPF6.3 (formerly NRT1.1) is highly coexpressed with H^+^-ATPase 2, AT4G30190 (Table 1 and Table S3). NPF6.3 is a nitrate/proton symporter, requiring a proton gradient for the uptake of nitrate from the soil (Parker and Newstead, 2014). The H^+^-ATPases that maintain the proton gradient comprise a large superfamily (Axelsen and Palmgren, 2001; Palmgren, 2001), but our coexpression analysis suggests that H^+^-ATPase 2, specifically, may contribute to the proton gradient needed for the transport of nitrate from the soil into the root by NPF6.3. Furthermore, H^+^-ATPase 2 is strongly expressed in the root pericycle, cortex, epidermis, and root cap according to the *Arabidopsis* eFP browser, particularly after nitrate addition (Figure S1A; Winter et al., 2007).

**Table 1:** **The top1 correlated genes for each of 17 N transporters**.

**NRT genes**	**Top 1 coexpressed genes**
CLCA	AT5G49360:beta-xylosidase 1
NPF2.7 (NAXT1)	AT5G43370:phosphate transporter 2
NPF6.3 (NRT1.1)	AT4G30190:H(+)-ATPase 2
NPF4.6 (NRT1.2)	AT4G33300:ADR1-like 1
NPF6.2 (NRT1.4)	AT2G45960:plasma membrane intrinsic protein 1B
NPF7.3 (NRT1.5)	AT3G23430:phosphate 1
NPF2.12 (NRT1.6)	AT2G22350:transposable element gene
NPF2.13 (NRT.17)	AT4G12280:copper amine oxidase family protein
NPF7.2 (NRT1.8)	AT5G13330:related to AP2 6l
NPF2.9 (NRT1.9)	AT4G34600:unknown
NRT2.1	AT4G32950:Protein phosphatase 2C family protein
NRT2.2	AT3G63100:unknown
NRT2.4	AT4G17710:homeodomain GLABROUS 4
NRT2.5	AT4G17710:homeodomain GLABROUS 4
NRT2.6	AT1G44930:unknown
NRT2.7	AT2G38210:putative PDX1-like protein 4
SLAH3	AT4G33420:Peroxidase superfamily protein

We further included 171 genes potentially involved in nitrogen transport in a similar analysis as above. A network of top 20 coexpressed partners for each nitrogen transporter can be visualized in Figure S2. In total, 2047 other genes are in this network, many of which are connected with more than one nitrogen transporter (Table S4). Interestingly, other transporter genes are enriched among those 2047 genes, such as genes from the GO categories “ion transport” and “carbohydrate transport” (Table S5), indicating those biological processes might be regulated similarly in *Arabidopsis* (Koprivova et al., 2000; Scheible et al., 2004). These other transporters could possibly be involved in transport of counter-ions to help maintain charge balance across membranes during sustained NO${}_{3}^{-}$ transport, or might be involved in the uptake or homeostasis of other essential nutrients that would be needed for growth and development at the same time as N uptake. Multiple other GO biological process categories were also significantly over-represented in the network (Table S5), such as those relating to phenolics. It is well documented that phenolic compound biosynthesis is upregulated when N is limited relative to C (Scheible et al., 2004; Cross et al., 2006). Our analysis suggests that there may be coordinated regulation of N transporter genes and phenylpropanoid biosynthetic genes. Additionally, there were various carbohydrate (C) metabolism and transport categories that were over-represented in the N transporter network (Table S5). This is consistent with previous evidence for extensive coordination to balance C and N metabolism (Palenchar et al., 2004) but may also indicate the need for increased carbohydrates in tissues where N uptake is strong to provide energy to maintain the proton gradient needed for N uptake, and to provide energy and organic building blocks for the lateral root proliferation that is common in high N regions of soil (Hodge, 2004). Finally, the network also included responses to numerous stimuli, such as water deficit, abscisic acid, and wounding. These “response” categories represent a rich resource for hypothesis generation, as they may reflect the importance of coordinating N utilization with other aspects of plant physiology in response to different environmental conditions. For example, N uptake may need to be altered if water uptake declines during drought, since N delivery to the shoot requires transport with water through the xylem. In addition to these over-arching insights, similar networks that focus on particular aspects of N transport (e.g., N export during leaf senescence) may be useful to identify a more focused set of processes that are associated with particular aspects of N utilization.

### Coexpression network indicates tissue specificity and potential pathways associated with N-transport

**Figure 3:**
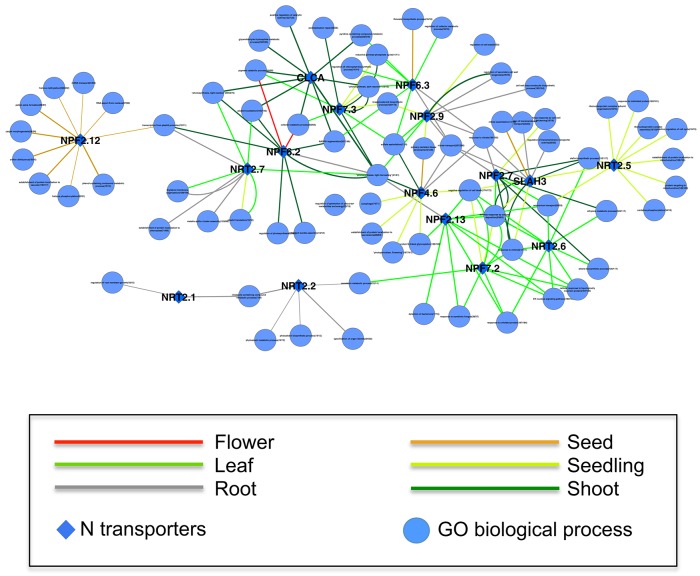
**A tissue-specific coexpression network between 17 N transporters and GeneOntology biological processes**. Only the top 10 statistically significant coexpressed pathways coexpressed with each N transporter were included. The numbers following the name of GO biological process represent the number of genes within the process/the number of genes within the process and are on the microarray. The width of the edge is corresponding to the average weight of coexpression in GEO datasets of a specific tissue between the N transporter and genes from the GO category. Only the edges supported by at least 5 datasets of a specific tissue are shown here. A high resolution version of this figure and all the weights between N transporter and GO biological processes in all available tissues can be found in Supplemental Materials (Figure S4 and Table S6).

Increasing the coexpressed partners in a network beyond the top 20, as above, may be meaningful but there is a risk of increasing the false-positive rate. One strategy to detect those broader relationships when individual gene relationships are relatively weak is to compute the correlation between a gene and meaningful pathways, such as GO Biological Processes (Huang et al., 2006; Tegge et al., 2012; Bateman et al., 2014). Since a pathway is a pre-defined group of genes, taking all those genes into account may boost the power to detect the real signal (Lee et al., 2011). Furthermore, the presence or absence of specific genes or networks may be tissue- or cell-type dependent (Anglani et al., 2014). We calculated the correlation between expression of the 17 N transporter genes and GO Biological Process pathways within each GEO dataset for samples from the same tissue type, and used the top 10 correlations to construct a network of N transporter-pathways that displays the tissue-specificity of each edge (See Section Materials and Methods; Figure 3). Each connection between a N transporter and a GO pathway represents a statistically significant correlation in a tissue type, which is represented by the color of the edge. Some N transporters are connected by network edges of a single tissue type, such as NPF2.12 (NRT1.6) and NRT2.2, while others are connected by network edges of multiple tissue types, such as NPF6.2 (NRT1.4) and NPF4.6 (NRT1.2) (Figure 3).

These correlations may provide hints as to the function of uncharacterized N transporter genes or additional functions of previously characterized genes. NPF2.12 (formerly NRT1.6) is connected with several pathways in our N transporter-pathway network and all those relationships are based on seed-specific datasets (Figure 3). This is consistent with previous evidence, which suggests that NPF2.12 is involved in the delivery of nitrate from the maternal plant to the developing embryo, particularly the transfer of nitrate from the vascular tissue into the seed (Almagro et al., 2008). Knockout of NPF2.12 has profound impacts such as reduced nitrate content of seeds, and substantially increased incidence of seed abortion. Our network suggests that, in addition to carpel development, NPF2.12 may also be strongly linked with anther and pollen development, vacuolar protein localization, and phenolic metabolism. In recent years, intact phenolic metabolism has been linked with proper pollen development and pollen fertilization of embryos (Matsuno et al., 2009; Fellenberg et al., 2012; Fellenberg and Vogt, 2015).

All of the edges connected to NRT2.2 are based on root-specific datasets, which is consistent with its role in the uptake of nitrate from soil (Li et al., 2007). One of the pathways connected to NRT2.2 is “specification of organ identity,” which might reflect the tight relationship between nitrate uptake and cellular differentiation or between nitrate consumption and root growth (Walch-Liu et al., 2006). NRT2.2 and NRT2.1 shared a strong link with “imidazole-containing compound metabolic process,” which includes multiple genes associated with histidine biosynthesis. Although the relevance of this co-linkage to histidine biosynthesis is not immediately clear, it is interesting that NRT2.2 and NRT2.1 are linked in our network since the two genes reportedly have some overlap of function in inducible high affinity nitrate uptake by roots (Li et al., 2007).

Table 2 shows the top correlated pathway for each of the 17 N transporters. Detailed information about all those tissue-specific correlations can be found in Table S6. We believe that data such as these will provide potential candidate genes and interesting hypotheses for further studies. For example, leaf-specific “sulfate (S) assimilation” is the best correlated pathway with NPF6.3 (formerly NRT1.1) and NPF7.3 (formerly NRT1.5). It is not surprising that N uptake and S assimilation genes correlate, since plant processes that require a lot of N also tend to need S, for example for the biosynthesis of cysteine, methionine, and several important cofactors (Koprivova et al., 2000). Similarly, multiple genes were best associated with, “photosynthesis, light harvesting,” including NPF4.6, NPF6.2 (formerly NRT1.2 and NRT1.4, respectively), NRT2.7, and CLCA, which appear as a cluster in the network (Figure 3). This may reflect the importance of tight co-regulation of N and C metabolism and also the fact that the biosynthesis of light harvesting proteins and pigments is highly dependent on the availability of N (Scheible et al., 2004).

**Table 2:** The top1 correlated pathways for each of 17 N transporters^*^.

**NRT genes**	**Tissue**	**Top 1 coexpressed GO biological process**
CLCA	Shoot	Photosynthesis, light harvesting
NPF2.7 (NAXT1)	Seed	Protein N-linked glycosylation
NPF6.3 (NRT1.1)	Leaf	Sulfate assimilation
NPF4.6 (NRT1.2)	Shoot	Photosynthesis, light harvesting
NPF6.2 (NRT1.4)	Shoot	Photosynthesis, light harvesting
NPF7.3 (NRT1.5)	Leaf	Sulfate assimilation
NPF2.12 (NRT1.6)	Leaf	Carpel morphogenesis
NPF2.13 (NRT.17)	Leaf	Negative regulation of cell death
NPF7.2 (NRT1.8)	Leaf	Defense response by callose deposition
NPF2.9 (NRT1.9)	Shoot	Regulation of secondary cell wall biogenesis
NRT2.1	Flower	Vesicle coating
NRT2.2	Leaf	Petal morphogenesis
NRT2.4	Leaf	Carpel morphogenesis
NRT2.5	Seed	Carpel morphogenesis
NRT2.6	Shoot	Ribosomal small subunit biogenesis
NRT2.7	Shoot	Photosynthesis, light harvesting
SLAH3	Seed	Nitrate assimilation

* Only GEO datasets of which all the samples are from the same tissue were used for this calculation.

Another example is NPF2.13 (formerly NRT1.7) which is coexpressed with “negative regulation of cell death” pathway in leaf. As reported previously, NPF2.13 plays a role in recycling of nitrogen within the plant (Fan et al., 2009) and cell death is a prominent event during senescence (Lim et al., 2007). It is probably crucial that cell death is slowed or delayed until most of the N is exported from the leaves through the phloem. Several other N transporter genes are also strongly correlated with the “negative regulation of cell death” pathway (NPF4.6/NRT1.2, NPF7.2/NRT1.8, NRT2.6, and NRT2.5), which might suggest that multiple N transporter genes are involved in N remobilization during senescence. NRT2.5 has been linked previously with N remobilization (Lezhneva et al., 2014). Alternatively, the “negative regulation of cell death” hub might indicate a general role of abundant N in delaying senescence. Indeed nitrogen status and senescence are known to be closely linked (Cooke et al., 2005; Diaz et al., 2008).

Some of the other GO Biological Process pathways appear to represent informative hubs. For example, the “nitrate transport,” and “response to nitrate,” groups are coexpressed with multiple genes in root tissues, including NPF6.3, NPF4.6, NPF2.9, NPF2.7, and SLAH3 (Figure 3). Several of these genes are known to be involved in nitrate uptake and redistribution in roots (Segonzac et al., 2007; Wang and Tsay, 2011; Glass and Kotur, 2013) and this association suggests that the others might play other roles in these processes. For example, SLAH3 functions in nitrate release from guard cells (Geiger et al., 2011), but based on the fact that SLAH3 is strongly expressed in the pericycle of roots (Figure S1B) combined with this coexpression relationship, one could hypothesize that SLAH3 might facilitate nitrate loading into the xylem or phloem in the roots via an apoplastic route. The role of NPF2.9 (formerly NRT1.9) was described as mediating nitrate distribution between shoot and root (Wang and Tsay, 2011). Based on expression of NPF2.9 in companion cells in roots and the relationship of NPF2.9 with NPF6.3 and NPF4.6 (formerly NRT1.1 and 1.2, respectively) in our coexpression network, perhaps NPF2.9 might have a more direct link with nitrate uptake, such as delivery of nitrate from the maturation zone of the root, where much of the water and nitrate uptake occurs, toward the developing root tip via the phloem. By identifying clusters or hubs such as this, our analysis provides guidance for further experimentation. For example, in order to understand nitrate uptake, we need to understand the functions of these genes and how these functions are integrated as a system.

## Discussion

One of the popular approaches to leverage expression data is coexpression network analysis. Transcriptome data probably is the most abundant biological data for plants, with more than 30,000 microarray samples deposited in NCBI GEO for the model plant *Arabidopsis* alone (He et al., 2016). This massive dataset is a valuable resource to functional genomics of plants. For example, genes involved in flavonoid biosynthetic process (Katsumoto et al., 2007), starch metabolism (Mentzen et al., 2008), aliphatic glucosinolate biosynthesis (Gigolashvili et al., 2009), lignin biosynthesis (Vanholme et al., 2013), and photorespiration (Pick et al., 2013) have been identified with the assistance of coexpression networks. Compared with animal data, functional gene annotation is limited in plants. It is critical to utilize the large amount of transcriptomics data to guide studies of gene function in plant science (Hwang et al., 2011). In fact, the standard gene annotations for *Arabidopsis* include data predicted based on coexpression networks (Heyndrickx and Vandepoele, 2012).

Generally speaking, large sample size helps to infer a more robust correlation relationship. If two genes show a high coexpression in only one dataset but very low coexpressions in other datasets, it may be a false positive due to the noise of microarray or stochasticity (Lee et al., 2004). Using integrated datasets helps to avoid those false positives, but may lead us to ignore biologically meaningful but transient patterns. Recently, the experimental evidence supporting the existence of transient relationships has been revealed (Ideker and Krogan, 2012). For example, a method called AP-SRM (Affinity Purification-Selected Reaction Monitoring) has been established to measure the physical interactions that only exist in certain conditions (Bisson et al., 2011). More than 70% of yeast genetic interactions under chemical treatment cannot be detected in a normal cellular environment (Bandyopadhyay et al., 2010). Plant scientists are also aware that coexpression networks are context dependent (Usadel et al., 2009). For instance, coexpressed partners of an *Arabidopsis* gene (i.e., RGL2) are highly dependent on the microarray samples used (Usadel et al., 2009). Instead of combining expression profiling samples from different labs, we calculated the strength of coexpression for each GEO dataset independently in order to capture those context-specific signals. As far as we know, our work is the first to perform context-specific coexpression analysis for genes involved in N transport. As the cost of RNAseq decreases, gene expression data will increase exponentially, and it will only become more crucial to have computational methods, such as those described here, to transform those vast amounts of data into refined hypotheses, and ultimately to expand our knowledge of plants as complex integrated systems.

## Conclusion

Here in our study, publicly available microarray data was utilized to explore the coexpression network of nitrogen transporters in *Arabidopsis*. A tight association between transport of nitrogen and other transport and metabolic processes was revealed. The co-regulated partners of N transporter genes was provided, serving as a resource for further studies. It is well known that carbon and nitrogen metabolism are tightly coordinated in plant tissues (Palenchar et al., 2004; Scheible et al., 2004; Cross et al., 2006). Our coexpression network supports the notion that there is coordination at the organismal level, and suggests that N transporters mediate at least some aspects of the coordination of C and N metabolism.

## Materials and methods

### Data collection and normalization

Three hundred and seventy one expression series datasets based on platform GPL198 were collected from GEO (Table S7). Each dataset contains at least 12 samples. Three hundred and twenty datasets which contain CEL files were used in our analysis. Robust Multiarray Average (RMA) was used to normalize the microarray data for each dataset (Irizarry et al., 2003). The IDs of probesets were converted into gene locus ID based on the annotation file for GPL198. The replicate group and tissue types were manually curated.

### Identification of differentially expressed genes

For each expression dataset, we applied ANOVA to identify gene expression which has larger variation between two replicate groups than within a replicate group. Only those with false discovery rate (FDR) < 0.001 were considered as DEG.

### Construction of coexpression network

We used the following equation to measure the strength of coexpression between a nitrogen transporter and another gene on the array:

(1) $$\begin{array}{l} R_{x,i}=\frac{\sum_{k=0}^{n}r_{k}}{n} \end{array}$$

where *r*_*k*_ is the coexpression weight (i.e., PCC) between gene *i* and nitrogen transporter *x* in the GEO dataset *n*. *R*_*x, i*_ is the average value of 320 weights between gene *i* and nitrogen transporter *x.* We used the following equation to measure the strength of tissue-specific coexpression between a nitrogen transporter and another gene on the array:

(2) $$\begin{array}{l} R_{x,i}^{{}^{\prime }}=\frac{\sum_{k\in T}^{n}r_{k}}{n} \end{array}$$

where *T* is the subset of GEO dataset of a specific tissue type. $R_{x,i}^{{}^{\prime }}$ is the strength of tissue-specific coexpression between a nitrogen transporter *x* and gene *i*. *r*_*k*_ in Equation (2) represents the weights from a specific tissue type, *T*. We used the “biological process” in the GeneOntology system to define a pathway and the following equation was used to measure the tissue-specific coexpression between a nitrogen transporter *x* and a pathway:

(3) $$\begin{array}{l} R_{x,p}^{{}^{\prime }}=\frac{\sum_{k\in p}^{m}R_{x,k}^{{}^{\prime }}}{m} \end{array}$$

where *m* is the number of genes in a pathway *p*. $R_{x,k}^{{}^{\prime }}$ is the tissue-specific coexpression between nitrogen transporter *x* and another gene *k*. And *k* is a gene in the pathway *p*. When we constructed the coexpression network between nitrogen transporters and pathways, only the datasets where a nitrogen transporter is differentially expressed were used. In order to determine the statistical significance of $R_{x,p}^{{}^{\prime }}$, the genes of a pathway were replaced by randomly selected genes in the genome and the $R_{x,p}^{{}^{\prime }}$ was calculated. We repeated this process 100 times for each pathway. A *empirical p* < 0.01 was assigned if none of the resulted $R_{x,p}^{{}^{\prime }}$ is higher than the real $R_{x,p}^{{}^{\prime }}$. For more detail, see Supplemental Note.

## Author contributions

FH, AK, SM, and BB conceived the study. FH performed analysis. FH, BB, and AK wrote the paper.

### Conflict of interest statement

The authors declare that the research was conducted in the absence of any commercial or financial relationships that could be construed as a potential conflict of interest.

## Supplementary material

Supplemental Note **Detailed information for the method of calculating *p-value* between a gene and a pathway**.

Figure S1 **Additional evidence of expression pattern for H_+_-ATPase 2 (A) and SLAH3 (B)**.

Figure S2 **The coexpression network of 171 N transporters**.

Figure S3 **The high-resolution version of Figure 2**.

Figure S4 **The high-resolution version of Figure 3**.

Table S1 **A list of nN transporters used in our coexpression analysis**.

Table S2 **Differential expression of 17 N transporters in roots and leaves**.

Table S3 **The coexpressed partners for each N transporters**.

Table S4 **Degree information for nodes in the coexpression network formed between 171 N transporters and their top20 coexpressed partners**.

Table S5 **Functional enrichment of coexpressed genes with 171 N transporters**.

Table S6 **Tissue-specific coexpressed biological processes with 17 N transporters**.

Table S7 **All GEO datasets used in this study**.
