# Supplementary material for: Large-Scale Public Transcriptomic Data Mining Reveals a Tight Connection between the Transport of Nitrogen and Other Transport Processes in Arabidopsis
Source: Front Plant Sci. 2016 Aug 11;7:1207. doi: 10.3389/fpls.2016.01207 (PMC4981021; doi:10.3389/fpls.2016.01207)
Supplement: Supplemental Note — Detailed information for the method of calculating p-value between a gene and a pathway. [file Presentation1.PDF]

In this note, we use an example to illustrate how we calculated the tissue-specific coexpression between a NRT (e.g NRT.17/NPF2.13) and a pathway (e.g 'ketone biosynthetic process') (see Table S6).

In the following figure, each number represents the coexpression weight, *R* between NPF2.13 and a gene from 'ketone biosynthetic process' in each GEO dataset, where NPF2.13 is detected as differential expression.

GSE datasets in which NRT1.7 is differentially expressed

42 genes in a GO group

	**GSE1**	**GSE2**	**...**	**GSEn**
**gene1**	*0.7*	*0.2*	...	*0.8*
**gene2**	*0.2*	*0.3*	...	*0.6*
**...**	...	...	...	...
**gene42**	*0.4*	*0.9*	...	*0.7*

Tissue: leaf

Tissue: root

For datasets of the same tissue type, the mean weight, *R'* was calculated between NPF2.13 and each gene of the 'ketone biosynthetic process'.

42 genes in a GO group

	**Leaf**	**flower**	**seed**	**seedling**	**root**
**gene1**	*0.34*	*0.31*	*-0.29*	*-0.09*	*0.51*
**gene2**	*0.19*	*-0.03*	*-0.46*	*0.53*	*0.27*
**...**	...	...	...	...	...
**gene42**	*0.88*	*0.12*	*0.18*	*0.26*	*-0.21*

Then, the average weight of all the genes in ‘ketone biosynthetic process’ of each tissue type was calculated. The number shown in the following table represents the tissue-specific coexpression weight between NPF2.13 and ‘ketone biosynthetic process’.

	leaf	root	seed	seedling	flower
NPF2.13	0.26	-0.29	0.31	0.24	0.23

In order to determine the significance of the coexpression between NPF2.13 and ‘ketone biosynthetic process’, we calculated the data based on randomly selected 42 genes from the genome. We repeated this process 100 times. If none of the coexpression weight from the random set of genes is higher than the real one, we consider *empirical p-value* < 0.01.
