# Supplementary material for: Large-Scale Public Transcriptomic Data Mining Reveals a Tight Connection between the Transport of Nitrogen and Other Transport Processes in Arabidopsis
Source: Front Plant Sci. 2016 Aug 11;7:1207. doi: 10.3389/fpls.2016.01207 (PMC4981021; doi:10.3389/fpls.2016.01207)
Supplement: Figure S1 — Additional evidence of expression pattern for H+-ATPase 2 (A) and SLAH3 (B). [file Image1.PDF]

Figure S1. Additional evidence of expression pattern for NPF6.3 and SLAH3

a) The expression pattern of AT4G30190 indicates it might be functionally associated NRT1.1/NPF6.3

AT4G30190 is the most strongly coexpressed gene with NRT1.1/NPF6.3 is strongly expressed in the root pericycle, cortex, epidermis, and root cap according to the Arabidopsis eFP browser, particularly after nitrate addition.

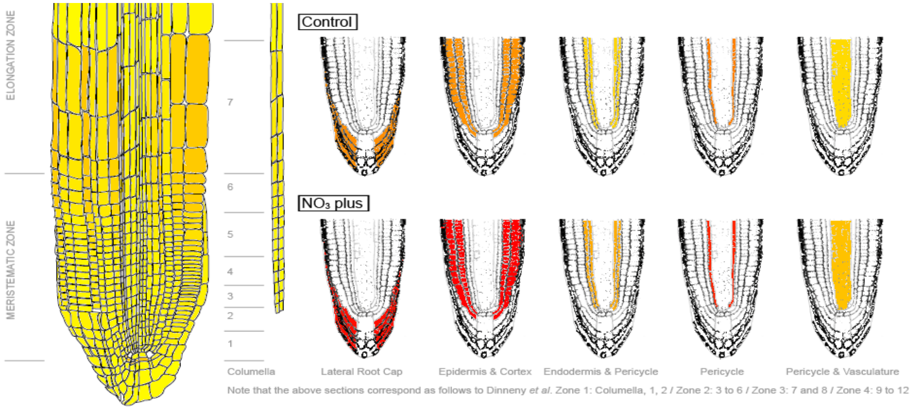

b) The expression pattern of SLAH3 indicates its role in root

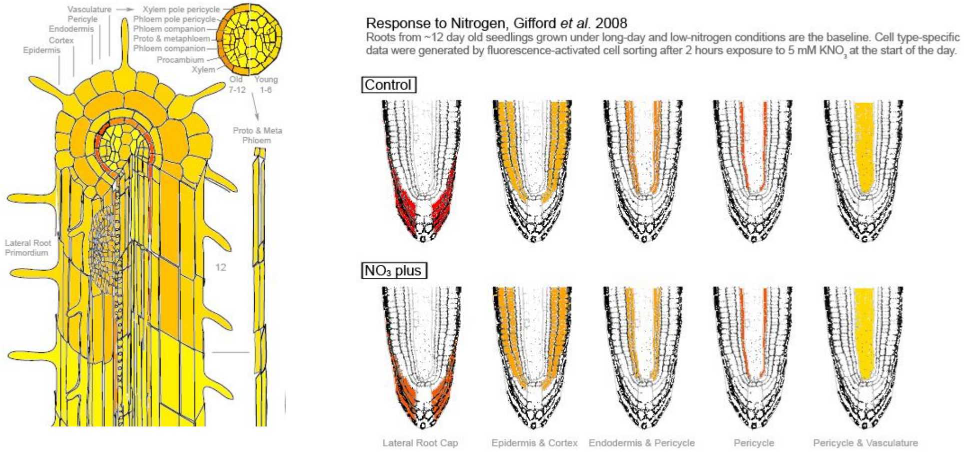

Pictures were generated by
http://bar.utoronto.ca/efp/cgi-bin/efpWeb.cgi
