# Supplementary material for: Large-Scale Public Transcriptomic Data Mining Reveals a Tight Connection between the Transport of Nitrogen and Other Transport Processes in Arabidopsis
Source: Front Plant Sci. 2016 Aug 11;7:1207. doi: 10.3389/fpls.2016.01207 (PMC4981021; doi:10.3389/fpls.2016.01207)
Supplement: Figure S2 — The coexpression network of 171 N transporters. [file Image2.PDF]

Figure S2

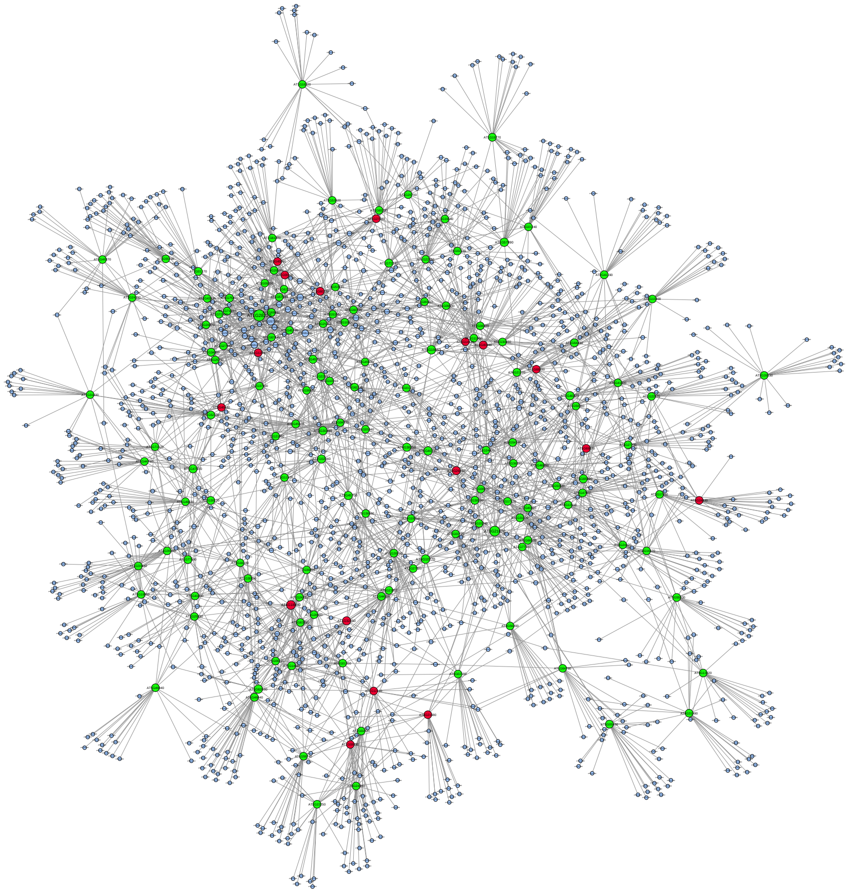

The 17 N transporters discussed in the main text are colored in red
Other N transporters in our collection of 171 genes are colored in green
Their top20 coexpressed genes are colored in blue
