# Supplementary figures and images for: Large-Scale Public Transcriptomic Data Mining Reveals a Tight Connection between the Transport of Nitrogen and Other Transport Processes in Arabidopsis
Source: Front Plant Sci. 2016 Aug 11;7:1207. doi: 10.3389/fpls.2016.01207 (PMC4981021; doi:10.3389/fpls.2016.01207)

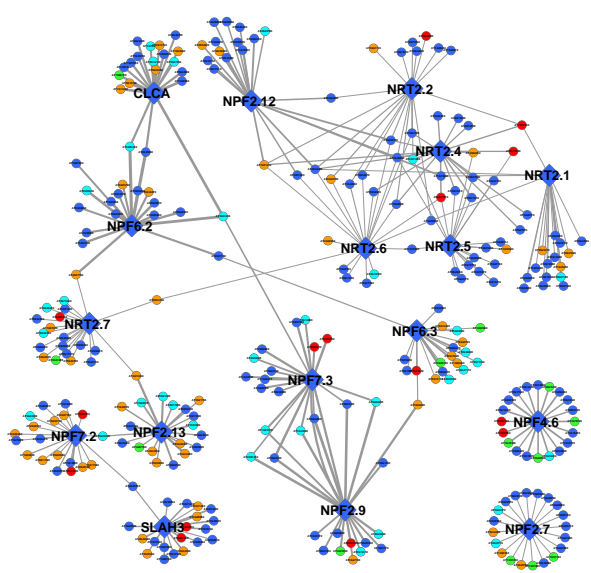

Supplement: Figure S3 — The high-resolution version of Figure 2. [file Image3.PDF]

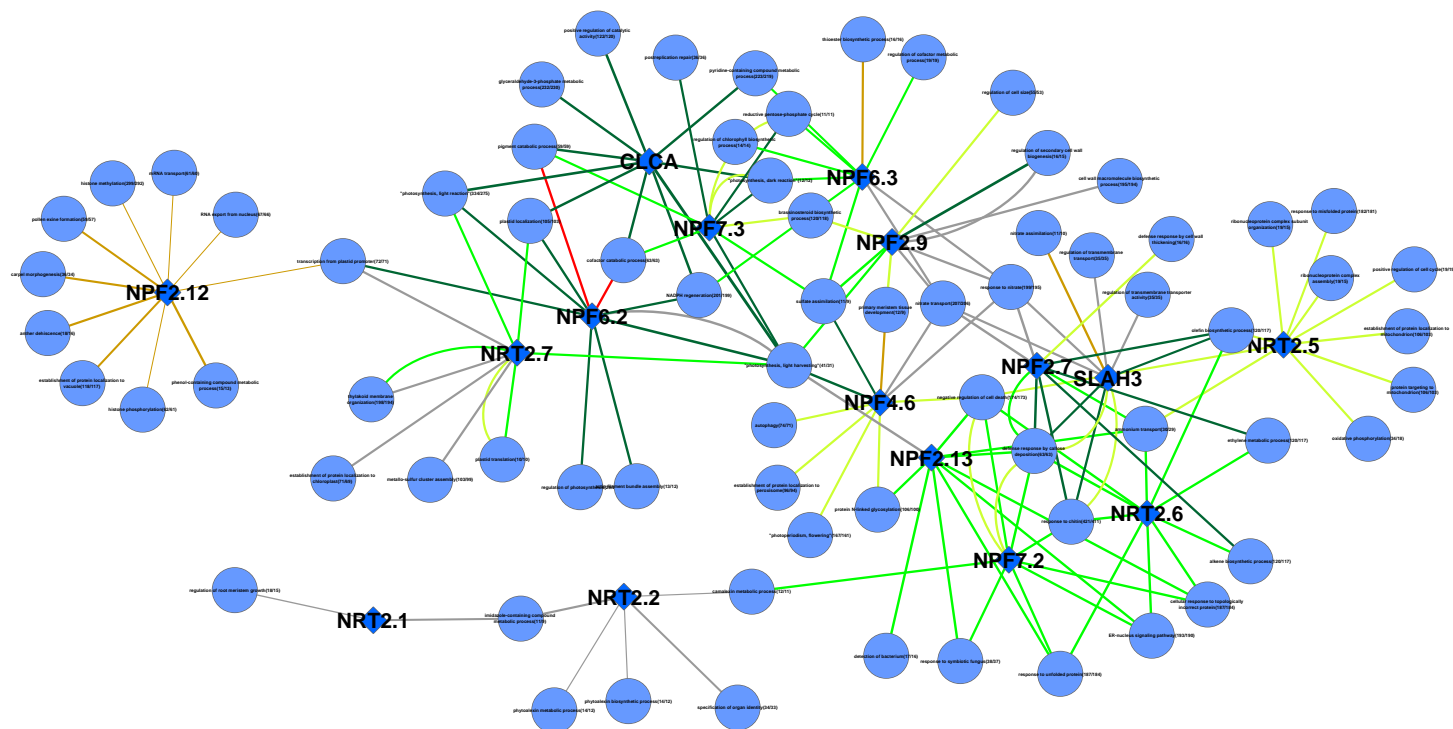

Supplement: Figure S4 — The high-resolution version of Figure 3. [file Image4.PDF]
